# Symptom clusters and related factors in bladder cancer patients three months after radical cystectomy

**DOI:** 10.1186/s12894-017-0255-x

**Published:** 2017-08-23

**Authors:** Hongyan Ren, Ping Tang, Qinghua Zhao, Guosheng Ren

**Affiliations:** 1 Department of Urology, The First Affiliated Hospital of Chongqing Medical University, No. 1 Youyi Road, Yuzhong District, Chongqing, 400016 China; 2 Department of Cardiology, The First Affiliated Hospital of Chongqing Medical University, No. 1 Youyi Road, Yuzhong District, Chongqing, 400016 China; 3 Department of Nursing, The First Affiliated Hospital of Chongqing Medical University, No. 1 Youyi Road, Yuzhong District, Chongqing, 400016 China; 4 Molecular Oncology and Epigenetics Laboratory, The First Affiliated Hospital of Chongqing Medical University, No. 1 Youyi Road, Yuzhong District, Chongqing, 400016 China

**Keywords:** Bladder cancer, Quality of life, Radical cystectomy, Symptom cluster, Symptom distress

## Abstract

**Background:**

To identify symptom distress and clusters in patients 3 months after radical cystectomy and to explore their potential predictors.

**Methods:**

A cross-sectional design was used to investigate 99 bladder cancer patients 3 months after radical cystectomy. Data were collected by demographic and disease characteristic questionnaires, the symptom experience scale of the M.D. Anderson symptom inventory, two additional symptoms specific to radical cystectomy, and the functional assessment of cancer therapy questionnaire. A factor analysis, stepwise regression, and correlation analysis were applied.

**Results:**

Three symptom clusters were identified: fatigue-malaise, gastrointestinal, and psycho-urinary. Age, complication severity, albumin post-surgery (negative), orthotropic neobladder reconstruction, adjuvant chemotherapy and American Society of Anesthesiologists (ASA) scores were significant predictors of fatigue-malaise. Adjuvant chemotherapy, orthotropic neobladder reconstruction, female gender, ASA scores and albumin (negative) were significant predictors of gastrointestinal symptoms. Being unmarried, having a higher educational level and complication severity were significant predictors of psycho-urinary symptoms. The correlations between clusters and for each cluster with quality of life were significant, with the highest correlation observed between the psycho-urinary cluster and quality of life.

**Conclusions:**

Bladder cancer patients experience concurrent symptoms that appear to cluster and are significantly correlated with quality of life. Moreover, symptom clusters may be predicted by certain demographic and clinical characteristics.

## Background

Radical cystectomy (RC) with urinary diversion is currently the standard treatment for muscle-invasive bladder cancer and is recommended for high-risk non-muscle-invasive bladder cancers. In China, bladder cancer is the eighth most common cancer in men and has recently increased in frequency [1]. Among diagnosed patients, 20% are candidates for RC [2]. However, bladder cancer itself and its treatment, such as surgery and chemotherapy, may cause symptom distress in patients, including fatigue, pain, appetite loss, nausea, vomiting, insomnia, diarrhea, colostomy problems, urine leakage, sexual dysfunction, and emotional distress [3–5]. Notably, clinical experiences have shown that multiple symptoms are clustered in cancer patients in disease trajectories [6]. Kim et al. [7] refined the definition of a symptom cluster (SC) as consisting of two or more symptoms that are related to each other and that occur together. The existence of SCs appears to exert a complex and synergistic detrimental effect on quality of life (QoL). Exploring the mechanics of concurrent symptoms such as SCs may facilitate the development of effective strategies to reduce the impact of treatment- and disease-related symptoms to improve patient QoL.

However, few studies have reported systematic, patient-reported symptom distress data, particularly at 3 months after operation, which is important for patient transition from post-surgery to long-term recovery. Based on previous studies, we hypothesized that patient symptoms might appear concurrently in a clustered manner and might be influenced by several demographic and disease variables. Therefore, the aims of this study were to (1) describe symptom experiences, (2) explore whether symptoms were clustered, (3) explore the potential predictors of each SC, and (4) analyze the correlations between SCs and QoL in bladder cancer patients 3 months after RC with an ileal conduit (IC) or orthotopic neobladder (ONB) reconstruction.

## Methods

### Patients and design

A cross-sectional design was used. The participants were consecutively enrolled from March 2013 to December 2015 at the first affiliated hospital of Chongqing medical university, China. The participant inclusion criteria included the following: (1) histologically confirmed bladder cancer and (2) treatment with RC combined with IC or ONB. The exclusion criteria included the following: (1) psychosis or cognitive impairment and (2) loss of contact or death during the study.

### Data collection

The clinical trial was approved by the university institutional review board. The demographic data were collected using an interview tool; the clinical information was collected by reviewing the patients’ medical records. Complications were defined as those occurring within 3 months after surgery. All events leading to a prolonged hospital stay or requiring medication or surgical intervention were scored as a complication. According to the Accordion Severity Grading System of surgical complications, complications were classified as minor, moderate or major.

The symptom questionnaires and the QoL assessments were completed through telephone or face-to-face interviews with the patients by trained oncology nurses 3 months after the operation. Data integration was reviewed immediately after the interviews, and any missing data were captured.

### Instruments

Patient symptoms were assessed using the revised symptom severity scale of the M.D. Anderson symptom inventory (MDASI) [8]. This original section of this scale has 13 items used to measure the severity of each symptom (item) in the previous 24 h, and the score ranges from 0 (“symptom not present”) to 10 (“as bad as you can imagine”). The Chinese version translated and tested by Wang et al. [9] was used. For our objectives, two disease-specific symptoms were added: one to measure urinary function (urinary control for ONB and urostomy management for IC) and the other to gauge patient dissatisfaction with one’s appearance. In the current study, the internal consistency coefficient (Cronbach’s alpha) for this 15-item scale was 0.803, and the content validity index determined by five urology experts was 0.855.

The QoL was measured using the self-administered Functional Assessment of Cancer Therapy-General (FACT-G) [10]; a higher score indicated a better QoL.

### Statistical analyses

Descriptive statistics were used to summarize the demographic and clinical characteristics and the prevalence and severity of symptoms. The SCs were identified by exploratory factor analysis using principal axis factoring. Similar to the method of Chen and Tseng [11], three items with inter-item correlations <0.40 (numbness, shortness of breath and dry mouth) were excluded from the factor analysis. Following the Kaiser-Meyer-Olkin measure (0.819) and Bartlett’s test of sphericity (*P* < 0.001), varimax rotation was used to simplify the factor structure, and the communality of remain items was 0.600 (standard deviation 0.120) which was adequately high to allow 99 samples to suffice for factor analysis. The internal reliability of each cluster was assessed by Cronbach’s alpha.

The association of SCs and QoL was determined using Pearson’s coefficient. Subsequently, each SC was regarded as a dependent variable, while the variables listed in Table 1 were regarded as independent variables. With an entrance criterion of 0.05 and a removal criterion of 0.1, stepwise multivariate linear regressions were applied to analyze which characteristics might be predictors of the independent variables. All tests were two-tailed, and *P* < 0.05 was considered significant. All analyses were performed using SPSS 20.0 software.

**Table 1:** Demographic and clinical characteristics of the participants

Characteristics	N (%) or Mean (SD)
Sex	
Male	93 (93.9%)
Female	6 (6.1%)
Cancer stage ^b^	
I/Tis	22 (22.2%)
II	64 (64.6%)
III	13 (13.1%)
Urinary diversion	
IC	67 (67.7%)
ONB	32 (32.3%)
Adjuvant chemotherapy	
No	54 (54.5%)
Yes	45 (45.5%)
ASA score ^a^	
1	51 (51.5%)
2	37 (37.4%)
3-4	11 (11.1%)
Postoperative complications, severity	
None	50 (50.5%)
Mild	21 (21.2%)
Moderate	19 (19.2%)
Severe	9 (9.1%)
Education level	
≤ 5 years	34 (34.3%)
6-11 years	50 (50.5%)
≥ 12 years	15 (15.2%)
Marital status	
Unmarried	24 (24.2%)
Married	75 (75.8%)
Albumin (mg/dl)	24.86 (5.00)
Age	61.85 (9.48)

^a^ASA score: American Society of Anesthesiologists score

^b^The total percentage is 99.9% with 0.1% error

## Results

### Patient characteristics

In total, 101 patients met the eligibility criteria, except two of them lost contacts, 99 patients participated in the study. As shown in Table 1, most patients were male, married, and had stage II bladder cancer.

### Symptom distress and SCs

The descriptive statistics regarding the symptom severity of the items and the prevalence of the symptoms 3 months after RC are presented in Table 2. The most severe symptom was distress, followed by fatigue and urinary dysfunction. Notably, pain ranked only eighth in symptom severity. Overall, all the participants experienced ten or more symptoms, and almost half of the participants (56.6%) reported more than four concurrent symptoms at moderate and severe levels.

**Table 2:** Descriptive statistics for symptom prevalence and severity

Symptoms	Severity Mean (SD)	Moderate ^a^ N (%)	Severe ^b^ N (%)
Distress	6.58 (1.43)	38 (38.4)	54 (54.5)
Fatigue	5.75 (1.77)	47 (47.5)	29 (29.3)
Urinary dysfunction	5.53 (1.38)	53 (53.5)	23 (23.2)
Loss of appetite	5.05 (1.66)	41 (41.4)	17 (17.2)
Sleep disturbance	4.74 (1.67)	43 (43.4)	12 (12.1)
Sadness	4.71 (1.21)	54 (54.5)	5 (5.1)
Body image dissatisfaction	4.62 (1.51)	40 (40.4)	9 (9.1)
Pain	4.14 (1.75)	30 (30.3)	7 (7.1)
Memory problems	4.10 (1.82)	27 (27.3)	10 (10.1)
Drowsiness	3.91 (1.90)	25 (25.3)	10 (10.1)
Nausea	3.62 (1.84)	17 (17.2)	8 (8.1)
Shortness of breath	2.17 (1.95)	9 (9.1)	4 (4.0)
Dry mouth	1.64 (2.21)	11 (11.1)	3 (3.0)
Vomiting	1.53 (1.17)	1 (1.0)	0
Numbness	1.46 (2.31)	8 (8.1)	5 (5.1)

^a^Score 5-6

^b^Score 7-10

Using an eigenvalue >1.0 as the criterion for retaining factors, a three-factor solution emerged, with 60.0% of the variance explained. Inspection of the items in each factor led us to name the clusters as the fatigue-malaise, gastrointestinal, and psycho-urinary SCs. Cronbach’s alpha for each of the clusters was 0.863, 0.625, and 0.701, respectively. The factor analysis results are shown in Table 3.

**Table 3:** Factor analysis of symptoms

Cluster	Factor Loading		
	Factor 1	Factor 2	Factor 3
Fatigue-malaise			
Fatigue	0.828		
Drowsiness	0.828		
Pain	0.811		
Memory problems	0.783		
Loss of appetite	0.536		0.484
Psycho-urinary			
Sleep disturbance		0.725	
Body image impairment		0.725	
Urinary dysfunction		0.654	
Sadness		0.646	
Distress		0.487	
Gastrointestinal			
Nausea			0.842
Vomiting			0.803
Explained variance	26.347	18.959	14.688
Cumulative variance	26.347	45.306	59.994

Extraction method: Principal component analysis. Rotation method: Varimax with Kaiser normalization

### Predictors of SCs

The results of the stepwise multiple regression analysis to predict SCs are presented in Table 4. Age, complication severity, plasma albumin after operation (negative), ONB, adjuvant chemotherapy and American Society of Anesthesiologists (ASA) score were significant predictors of fatigue-malaise distress and explained 38.5% of the total variance. Adjuvant chemotherapy, ONB, female gender, ASA score and albumin (negative) were significant predictors of gastrointestinal distress and explained 33.4% of the total variance. Being unmarried and having a higher educational level and complication severity level were significant predictors of psycho-urinary distress and explained 24.0% of the total variance.

**Table 4:** Stepwise regression model of predictors for symptom clusters

Model	*B*	SE	*Sta. B*	*t*	*P*	*Adj R* ^*2*^
Fatigue-malaise						
(Constant)	10.209	5.298		1.927	0.057	
Age	0.257	0.065	0.341	3.944	0.000	0.133
Complication severity	1.084	0.604	0.156	1.794	0.076	0.221
Albumin	- 0.408	0.120	−0.278	−3.394	0.001	0.273
Urinary diversion(0 = IC, 1 = ONB)	3.716	1.308	0.244	2.842	0.006	0.311
Adjuvant chemotherapy (0 = no, 1 = yes)	3.138	1.143	0.220	2.744	0.007	0.346
ASA score	2.355	0.894	0.225	2.636	0.010	0.385
Gastrointestinal						
(Constant)	4.421	1.299		3.404	0.001	
Adjuvant chemotherapy (0 = no, 1 = yes)	2.511	0.438	0.478	5.740	0.000	0.178
Urinary diversion (0 = IC, 1 = ONB)	1.614	0.472	0.288	3.420	0.001	0.233
Sex (0 = male, 1 = female)	2.312	0.924	0.211	2.503	0.014	0.274
ASA score	0.829	0.319	0.216	2.599	0.011	0.310
Albumin	−0.094	0.045	−0.173	−2.086	0.040	0.334
Psycho-urinary						
(Constant)	24.918	1.627		15.312	0.000	
Marital status (0 = unmarried, 1 = married)	−3.884	1.019	−0.343	−3.813	0.000	0.154
Education level	1.893	0.644	0.264	2.938	0.004	0.213
Complication severity	0.879	0.419	0.185	2.097	0.039	0.240

### Correlation of SCs with QoL

As shown in Table 5, the mean score of psycho-urinary problems was higher than that of fatigue-malaise and gastrointestinal symptoms. According to the percentage of the total score, the QoL score (50.39 **±** 12.39) was low. The correlations between clusters and each cluster with quality of life were significant, and the maximum correlation observed was between psycho-urinary symptoms and QoL (*r* = 0.685), while the minimum correlation observed was between psycho-urinary symptoms and gastrointestinal symptoms (*r* = 0.268).

**Table 5:** Bivariate correlations between symptom clusters and QoL

Variable	Mean (SD)	Fatigue-malaise	Gastrointestinal	Psycho-urinary
Fatigue-malaise (0-50)	22.95 (7.15)	1.00		
Gastrointestinal (0-20)	5.14 (2.63)	0.398^**^	1.00	
Psycho-urinary (0-50)	26.16 (4.88)	0.429^**^	0.268^**^	1.00
QoL (0-108)	50.39 (12.39)	−0.595^**^	−0.311^**^	−0.685^**^

**: *P* < 0.01

## Discussion

This study revealed that patients with RC experienced multiple symptoms simultaneously 3 months after RC from which three significant SCs were derived: fatigue-malaise, gastrointestinal, and psycho-urinary symptoms. Compared with the most commonly reported SCs from a systematic review, notwithstanding slight differences in the composition of items, the core symptoms tended to remain the same, such as fatigue, drowsiness and pain for fatigue-malaise, nausea and vomiting for gastrointestinal, and anxiety and depression for psycho-urinary symptoms [12]. The difference may be due to incongruous used scales, analytical techniques or the disease stage of the objects, and suggested that the SC composition or prevalence may change with the cancer type and stage.

When interpreting the results of factor analysis, consideration of the underlying commonality of the symptoms is important because one symptom may be a cause of another symptom or the symptoms may share a cause [13]. Therefore, we further explored the possible related factors. These findings will aid our understanding of the symptoms experienced by patients and facilitate further patient-centered symptom management in advanced bladder cancer for the period of transitional care from the prolonged peri-operative phase to long-term rehabilitation.

### Fatigue-malaise

The fatigue-malaise SC consisted of non-specific symptoms, including fatigue, drowsiness, pain, memory problems and lack of appetite. It is similar to the “sickness cluster” reported by Chen and Tseng [11], with which four items were found in common (i.e., fatigue, pain, lack of appetite, and drowsiness). We also found that this cluster is well matched to the concept of systemic exertion intolerance disease (SEID), which was defined as a substantial reduction or impairment in the ability to engage in pre-illness levels of occupational, educational, social, or personal activities; post-exertional malaise and unrefreshing sleep; and at least one of the following two symptoms: cognitive impairment and orthostatic intolerance [14].

Researchers have attempted to explain the mechanism of this cluster. Chen and Tseng [11] observed that this SC is mediated by proinflammatory cytokines (e.g., interleukin-1, interleukin-6, TNF-α, and interferon-α). In this study, proinflammatory cytokines and hypothalamic-pituitary-adrenal (HPA) function were not directly measured; however, the regression analysis revealed that physiological factors, such as postoperative complications, albumin and ASA scores, were significant predictors of such symptom distress. These factors also provided evidence supporting the conceptual meaning of the “fatigue-malaise cluster”.

### Nausea-vomiting

Similar to previous studies, nausea and vomiting combined to form an independent and robust cluster [8, 13, 15]. Although Cronbach’s coefficient was only 0.625, this value was acceptable considering the underlying commonality; however, whether loss of appetite was involved remains debatable. In some studies, such as the study by Sarna and Brecht [13] on lung cancer, loss of appetite was combined with nausea-vomiting. In the present study, loss of appetite overlapped with both the fatigue-malaise and nausea-vomiting clusters, while the loading coefficient was lower for the nausea-vomiting cluster than the former. As indicated by Molassiotis et al. [16], the presence of a symptom in more than one cluster is clinically meaningful, contributes to an increase in the internal consistency of the cluster, and may potentially indicate the biological outcome or other link between the two clusters. Indeed, the overlapping of loss of appetite in the gastrointestinal and fatigue-malaise clusters reflects the link between these two clusters.

The regression analysis showed that adjuvant chemotherapy, ONB surgery, female gender, and ASA scores significantly increased the burden of nausea and vomiting. Nausea and vomiting are generally considered side effects of chemotherapy. Additionally, female gender is well established as the most important predictor of postoperative- and chemotherapy-related nausea and vomiting [17, 18]. Nevertheless, in this study, ONB surgery was also shown to be a predictor of nausea and vomiting. Published studies have compared ONB and IC surgeries using a variety of indices; however, to date, no data account for this gastrointestinal symptom difference in such a transitional phase. Conditions associated with this difference may include a longer operative time and more complications of prolonged ileus obstruction after ONB surgery. Additionally, in accordance with Sarna and Brecht [13] we found that the comorbidities of systemic disease (ASA scores) significantly increase the distress of nausea-vomiting. Although gastrointestinal motility disorders associated with diabetes mellitus primarily due to autonomic nervous dysfunction have been well established [19], the evidence of an association of nausea and vomiting with other systemic diseases, such as cardiovascular disease, has not been determined. Rather, patients with a longer duration of illness are more likely to be poly-symptomatic, which may partly explain this finding [20].

### Psycho-urinary

Distress, sadness, urinary dysfunction, body image, and sleep disturbance comprised the psycho-urinary cluster. The grouping of distress and sadness as a cluster in cancer patients has already been shown in several studies [11, 21]. However, in this study, impaired urinary function, body image dissatisfaction, and sleep disturbance were highly linked to emotional symptoms and comprised a cluster considered unique to patients with RC. In accordance with the results of Punnen et al. [22], when urinary function worsened, individuals experienced more body image impairment and depressive symptoms. Furthermore, due to a decrease in the tone of the external sphincter, the urethral pressure is reduced during sleep, causing more severe nighttime urinary dysfunction, which could exacerbate sleep disturbance, emotional distress, and sadness. Zahran et al. [23] also compared people with and without nocturnal incontinence, and the results showed that the latter group had worse emotional and social function than the former group. The results suggest that urinary function is an important factor to consider in symptom management for depression.

Two demographic factors, educational level and marital status, correlated with the severity of psycho-urinary cluster symptoms. As reported by Xiao et al. [24], a positive relationship between a higher education level and symptom distress was found; moreover, as they discussed, patients who are more highly educated may seek more health care assistance and have a higher potential and willingness to report more symptoms. Sarna and Brecht [13] also reported that distress was associated with the educational level, with the greatest distress found among those with the most education. In this study, the patients with a higher education may have had higher expectations regarding the therapeutic effects and their QoL; therefore, they might have been more sensitive to psycho-urinary symptoms at this early rehabilitation stage. The relationship between marital status and psycho-urinary symptoms may be due to the intermediate function of social support, including medical help and emotional support, which was previously confirmed to be positively related to disease outcome in other chronic diseases [25].

### Correlation of SCs and QoL

The results of this study further confirmed that symptom distress was a reliable predictor of QoL. Greater symptom distress resulted in a poor QoL. Sun et al. [26] demonstrated that the symptom experience, such as nausea, appetite, and insomnia, significantly impacted QoL, particularly aspects of health and psychosocial behavior in bladder cancer patients. The possible SCs and their impact on QoL have also been explored in other diseases. Hwang et al. [21] reported that in ovarian cancer patients, the SCs of abdominal discomfort, psychological distress, fatigue-pain, and flu-like symptoms explained 46.0%, 40.0%, 38.0%, and 37.0% of the total variance in QoL, respectively, which partially supports our results. However, Somani et al. [27] systematically reviewed the association between body image distress and QoL and obtained different results, which suggests that this factor may not always be important for the QoL of RC patients. These contradictory results may be due to the different post-RC study period investigated. Our research targeted the early rehabilitative period, which was characterized by adaptation and adjustment, and during this period, QoL was worse and influenced by many factors.

Among the three clusters, the psycho-urinary cluster had the strongest association with QoL. This result is in agreement with a previous study by Kim et al. [28], which indicated that psychological distress was the most significant predictor of QoL and explained 20.1% of the total variation. Dong et al. [29] found that the emotional cluster (tense-worried-irritable-depressed) was a stronger predictor of overall QoL compared with other clusters, and this result reinforces the significant effect of psycho-urinary symptoms on QoL.

It should be noted the sexual symptom didn’t assessment in this research because the response rate was too low during the preliminary experiment. Further inquiry revealed the attitude of Chinese to it were much conservative, since most of them neither had sexual activity before surgery nor believed such activity was applicable during such stage of disease.

This study has some limitations. First, this was a single-centered research study that occurred in China and notwithstanding a consecutive enrolling method, the female enrollment rate was very low. Second, except for IC and ONB, other methods of urinary diversion such as cutaneous ureterostomy, for which comparable peri-operative outcomes in high-risk older adults were recently reported [30], were not included in this study. Therefore, the results must be carefully interpreted for females and patients who undergo other types of urinary diversion.

## Conclusion

Based on the aforementioned results, medical staff should be aware of the symptom management of patients with bladder cancer 3 months after RC and focus on three areas: fatigue-malaise improvement, nausea-vomiting control, and psycho-urinary adjustment. The fatigue-malaise management should begin with the diligent recognition and treatment of postoperative complications and comorbidities, along with the prevention of malnutrition. Nausea-vomiting management should be focused more on females and those with ONB surgery and comorbidities. Besides, the most important goal should be emotional adjustment and urinary function restoration. Since urinary incontinence or urostomy management and emotional disorders coexist within the same cluster, the diagnosis and management of any symptom in this cluster should not be formulated in isolation. A combination of strategies that target both aspects in a cluster should be administered simultaneously or in an orderly manner, while the demographic and clinical characteristics should be considered to relieve symptom distress and improve QoL in this transitional phase.

## Abbreviations

ASA: American Society of Anesthesiologists
IC: Ileal conduit
MDASI: M.D. Anderson symptom inventory
ONB: Orthotropic neobladder
QoL: Quality of life
RC: Radical cystectomy
